# Maternal Diet Associated with Oligosaccharide Abundances in Human Milk from Latina Mothers

**DOI:** 10.3390/nu16121795

**Published:** 2024-06-07

**Authors:** Pari Mokhtari, Kelsey A. Schmidt, Hashem Zamanian, Mahsa Babaei, Christopher J. Machle, Diana Trifonova, Tanya L. Alderete, Elizabeth A. Holzhausen, Jonatan Ottino-González, Bridget N. Chalifour, Roshonda B. Jones, Annalee Furst, Chloe Yonemitsu, Lars Bode, Michael I. Goran

**Affiliations:** 1 Department of Pediatrics, The Saban Research Institute, Children’s Hospital Los Angeles, Los Angeles, CA 90027, USA; pmokhtari@chla.usc.edu (P.M.); kelschmidt@chla.usc.edu (K.A.S.); mbabaie@chla.usc.edu (M.B.); cmachle@chla.usc.edu (C.J.M.); jongonzalez@chla.usc.edu (J.O.-G.); 2 The Saban Research Institute (TSRI) Data Science, Children’s Hospital Los Angeles, Los Angeles, CA 90027, USA; hzamanian@chla.usc.edu; 3 Department of Integrative Physiology, University of Colorado Boulder, Boulder, CO 80309, USA; ditr2320@colorado.edu (D.T.); elizabeth.holzhausen@colorado.edu (E.A.H.); bridget.chalifour@colorado.edu (B.N.C.); 4 Department of Environmental Health and Engineering, Johns Hopkins Bloomberg School of Public Health, Baltimore, MD 21205, USA; taldere1@jhu.edu (T.L.A.); rosjones@chla.usc.edu (R.B.J.); 5 Department of Pediatrics, Larsson-Rosenquist Foundation Mother-Milk-Infant Center of Research Excellence (MOMI CORE), and the Human Milk Institute (HMI), University of California, San Diego, La Jolla, CA 92123, USA; aloeffler@ucsd.edu (A.F.); chloeyonemitsu@yahoo.com (C.Y.); lbode@health.ucsd.edu (L.B.)

**Keywords:** human milk oligosaccharides, mother, lactation, breastfeeding, diet, micronutrients

## Abstract

Growing evidence indicates that human milk oligosaccharides (HMOs) are important bioactive compounds that enhance health and developmental outcomes in breastfed babies. Maternal dietary intake likely contributes to variation in HMO composition, but studies identifying diet–HMO relationships are few and inconsistent. This study aimed to investigate how the maternal intake of macronutrients and micronutrients—specifically proteins, fats, vitamins, and minerals—associated with HMOs at 1 month (n = 210), 6 months (n = 131), and 12 months postpartum (n = 84). Several associations between maternal dietary factors and HMO profiles were identified utilizing partial correlation analysis. For example, maternal free sugar (rho = −0.02, *p* < 0.01), added sugar (rho = −0.22, *p* < 0.01), and sugary sweetened beverage (rho = −0.22, *p* < 0.01) intake were negatively correlated with the most abundant HMO, 2′-fucosyllactose (2′-FL), at 1 month, suggesting that higher sugar consumption was associated with reduced levels of 2′-FL. Further, vitamins D, C, K, and the minerals zinc and potassium were positively correlated with 2′-FL at 1 month (p_All_ < 0.05). For the longitudinal analysis, a mixed-effects linear regression model revealed significant associations between maternal vitamin intake and HMO profiles over time. For example, for each unit increase in niacin intake, there was a 31.355 nmol/mL increase in 2′-FL concentration (*p* = 0.03). Overall, the results provide additional evidence supporting a role for maternal nutrition in shaping HMO profiles, which may inform future intervention strategies with the potential of improving infant growth and development through optimal HMO levels in mothers’ milk.

## 1. Introduction

Human milk is the optimal nutrition for healthy term infants, recommended exclusively for the first 6 months and as a key calorie source through the first two years [1]. This recommendation stems from extensive evidence highlighting the association between human milk consumption with a lower prevalence of chronic diseases such as obesity, diabetes, and cardiovascular disease [2,3], as well as improved developmental outcomes [4]. Human milk not only contains vital macronutrients and micronutrients but also bioactive components that may contribute to the health benefits of human milk. Human milk oligosaccharides (HMOs), a group of indigestible carbohydrates and the third most abundant solid constituent of human milk after lactose and lipids [5], have been shown to contribute to the observed health benefits of human milk consumption, including infant weight and body composition [6,7,8], immune function [9], and gut microbiome and brain development [10,11,12,13].

HMO biosynthesis begins with an elongated lactose molecule that is either fucosylated or sialylated, resulting in a diverse range of HMOs with varied functional properties and biological potential [14]. HMO composition is largely driven by genetics, where women with an active secretor locus encoding a functional fucosyltransferase 2 enzyme produce higher concentrations of alpha-1-2-fucosylated HMOs like 2′-fucosyllactose (2′-FL) or lacto-N-fucopentose I (LNFP I) relative to those classified as non-secretors [15,16,17]. Breastfeeding exclusivity and parity have also been identified as drivers of individual HMOs and contribute to differences in HMO profiles between mothers [16]. Consistent with our previous findings, HMO composition varies within women over the course of lactation, with the majority of HMOs decreasing in concentration between 1 and 24 months postpartum, while the HMOs, 3-fucosyllactose (3′-FL), and 3′sialyllactose (3′-SL) increase in concentration. Additionally, 2′-FL remains stable and does not change significantly over time [18]. However, the influence of modifiable lifestyle factors on HMO profiles, such as maternal diet, remains under-explored.

Only a few studies have investigated the relationship between maternal diet and HMO profiles, with mixed results. Azad et al. found that dietary factors were not associated with individual HMO concentrations at 3–4 months of lactation, although fucosyl-LNH (FLNH), lacto-N-tetraose (LNT), sialyl-LNT b (LSTb), and difucosyl-LNT (DFLNH) were marginally associated (*p* < 0.05) with individual food groups [16]. In a longitudinal cohort study, Quin et al. reported positive associations of maternal whole fruit and dietary fiber with HMO-bound fucose in maternal milk [19]. In a cross-over trial by Seferovic et al., mothers were randomized to glucose or galactose as their sole carbohydrate source for 30–57 h and carbohydrate or fat as their main energy source over eight consecutive days. There were no significant differences in the concentrations of individual HMOs, but there was a significant decrease in HMO-bound fucose in response to glucose vs. galactose and an increase in HMO-bound sialic acid in response to carbohydrate vs. fat [20]. Therefore, most studies conclude that maternal diet likely influences the HMO profile, though the diet–HMO relationships identified are inconsistent.

While there is preliminary evidence indicating an association between maternal diet and HMO profiles, more data are needed to confirm these relationships among a diverse range of study populations and during specific periods of lactation. Further, to our knowledge, no study has examined the associations of maternal micronutrient intake on the HMO profile in Latinas. Therefore, this study aimed to analyze the relationship between maternal diet, including macronutrient and micronutrient intake, and HMO profiles using data from an established cohort of Latino infant–mother dyads in Southern California at 1 month, 6 months, and 12 months postpartum. A further exploration of how maternal diet is associated with HMO abundances in human milk is essential, particularly in addressing the current literature gap concerning dietary factors’ impact on HMOs. This knowledge is key to developing targeted dietary intervention strategies to influence the HMO profile, which may confer health benefits to the infant.

## 2. Materials and Methods

### 2.1. Study Participants

We obtained data from the Southern California Mother’s Milk Study, which is an ongoing longitudinal cohort study of Latino mother–infant dyads, as reported previously [21]. Briefly, participants were eligible if they (a) self-identify as Hispanic/Latino (both mother and father), (b) had a singleton birth, (c) intended to breastfeed for at least 3 months, (d) enrolled within 1 month of the infant’s birth, and (e) were able to understand the procedures of the study and read English or Spanish at the fifth-grade level. Participants were not eligible if they (a) had a physician diagnosis of a major medical illness or an eating disorder, (b) had a physical, mental, or cognitive issue that prevented participation, (c) reported chronic use of medication that may affect body weight or composition, insulin resistance, or lipid profiles, (d) were a current smoker or were a user of other recreational drugs, (d) had a pre-term/low-birth weight infant or diagnosis of any fetal abnormalities, or (e) were less than 18 years old. Institutional review boards at the University of Southern California and Children’s Hospital Los Angeles approved this study (Protocol ID: CHLA-18-00576). Written informed consent was obtained from all mothers prior to data collection.

### 2.2. Demographics and Anthropometrics

Maternal body mass index (BMI, kg/m^2^) was measured using standardized height and weight assessments conducted by trained healthcare professionals. Additionally, demographic characteristics and relevant breastfeeding practices, including age and the number of breastfeeds per day, were collected through questionnaires.

### 2.3. Dietary Assessment

Trained bilingual research personnel conducted 24 h dietary recalls, including 2 weekdays and 1 weekend day, with mothers over the phone at baseline and when infants were 6 months and 12 months of age. These recalls involved detailing the food and drinks consumed by the mothers during the preceding 24 h period. These assessments were conducted to track changes in the mothers’ diet over time. Dietary intake at 6 and 12 months postpartum was measured based on the average from multiple 24 h dietary recalls. Mothers were interviewed to provide detailed dietary recalls, including portion sizes, brand names, and preparation methods. They described their dietary intake during the previous 24 h period. Before completing the recalls, the research team provided instructions on how to estimate food and beverage portion sizes, and mothers were provided with portion size information booklets. The procedures used in the 1986–1989 United States Department of Agriculture Continuing Survey of Food Intakes of Individuals were followed, and all recalls were collected in a personal interview using a standardized “multiple pass” protocol [22]. Dietary intake analysis was performed using the Nutritional Data System for Research software (NDSR) (University of Minnesota, version 2014–2019) [23,24]. Nutrient intake data, including macronutrients (carbohydrates, proteins, fats, and fibers) and micronutrients (vitamins and minerals), were extracted from the dietary recall data using the NDSR software. For this study, we examined the associations between gram intakes of dietary sugar (total, free, and added sugar) and fiber (soluble and insoluble fiber) from all sources at 1, 6, and 12 months postpartum. Sugars were categorized into three types based on guidelines from the World Health Organization (WHO) and the nutrient analysis software NDSR [25]. Free sugars include sugars naturally found in honey, syrups, and fruit juices, as well as those added to foods and beverages. Added sugars refer to sugars added to foods and drinks during manufacturing, cooking, or processing, excluding naturally occurring sugars such as those in fruit juice. Total sugars encompass all sugars present in a food or drink, whether naturally occurring or added, including free sugars, intrinsic sugars, and milk sugars.

Data from the 24 h dietary recalls were analyzed using NDSR to determine total and added sugar intake. To estimate free sugar intake, the research team separately analyzed sugars in beverages and non-beverages. For beverages, the total sugar content was adjusted by subtracting lactose, whereas for non-beverages, only the added sugars were classified as free sugars. These values were then aggregated to determine the total free sugar intake for each participant.

### 2.4. Human Milk Sample Collection

Human milk was obtained following a minimum interval of 1.5 h after the last feeding, and the mothers observed a fasting period of at least 1 h. In our clinical facility, participants underwent a single complete breast expression from the right breast, utilizing an electric breast pump, which guaranteed the collection of foremilk, midmilk, and hindmilk, as detailed in a previous study [18,26]. The collected human milk was subsequently blended, divided into 10 separate 500 μL tubes, and any remaining milk was placed in 5 mL tubes before being stored at a temperature of −80 °C.

### 2.5. HMO Measurements

For all HMO assays, raffinose was added to each sample as an internal standard for absolute quantification. HMOs were isolated with high-throughput solid-phase extraction, fluorescently labeled, and measured using high-performance liquid chromatography [10]. Nineteen HMOs were quantified based on standard retention times and mass spectrometric analysis. These individual HMOs account for >90% of total HMO composition and include the following: 2′-fucosyllactose (2′-FL), 3-fucosyllactose (3′-FL), 3′-sialyllactose (3′-SL), 6′-sialyllactose (6′-SL), difucosyllactose (DFLac), lacto-N-tetraose (LNT), lacto-N-neotetraose (LNnT), lacto-N-fucopentaose I (LNFP I), lacto-N-fucopentaose II (LNFP II), lacto-N-fucopentaose III (LNFP III), sialyl-LNT b (LST b), sialyl-LNT c (LST c), difucosyl-LNT (DFLNT), disialyllacto-N-tetraose (DSLNT), lacto-N-hexaose (LNH), fucosyl-LNH (FLNH), difucosyl-LNH (DFLNH), fucosyl-disialyl-LNH (FDSLNH), and disialyl-LNH (DSLNH).

### 2.6. Statistical Analysis

In this analysis, we had available data from 210 participants at 1 month of infant age, 131 participants at infant 6 months of age, and 84 participants at infant 12 months of age. Descriptive statistics are presented as mean ± standard deviation (SD) or as frequency (percentage) for categorical variables. Before assessing potential mean differences, we conducted normality and homoscedasticity tests for all variables to evaluate whether they met the assumptions required for the Analysis of Variance (ANOVA). Additionally, we performed Levene’s test to assess the homogeneity of variances before conducting the ANOVA. For variables meeting the assumptions of normality and homoscedasticity, the ANOVA was utilized. Conversely, for variables not meeting these assumptions, we employed the Kruskal–Wallis test as a non-parametric alternative.

A partial correlation analysis, utilizing the Spearman method, was performed to examine the independent associations between maternal diet, including macronutrient and micronutrient intakes, on HMO profiles among Latino women at 1 month, 6 months, and 12 months postpartum. This analysis method was chosen to examine the associations between the selected variables while controlling for potential confounding factors. The analysis included five covariates, adjusting for mother’s BMI, the infant’s age in days, the mother’s age, the mother’s energy intake, and secretor status. Socioeconomic status, as measured by Hollingshead index of socioeconomic status [27], was considered as an additional covariate but was ultimately excluded from the analysis as the inclusion of this factor did not make significant changes to the results. To control the False Discovery Rate (FDR), *p*-values were adjusted using the Benjamini–Hochberg method, setting a threshold for significance at 0.05.

A longitudinal assessment of the associations between maternal diet on the HMOs 2′-FL, 3′-FL, and 3′-SL was conducted using a mixed-effects linear regression model. Prior to conducting the regression analysis, an exploratory analysis was carried out to identify significant changes in dietary variables over the three time points: 1 month, 6 months, and 12 months. Significant variations in dietary components were observed at each time point. Variables with *p*-values less than 0.05 were considered significant and included in the subsequent regression analysis. Individual HMOs were regressed on a set of predictor variables (dietary variables) along with ‘time point’ as an additional fixed effect. The model also included a random slope term for time, which allows for the relationship between HMOs and time to vary across individuals. This approach allowed us to account for both the fixed effects of diet and time as well as the random effects associated with individual differences among participants. The model was adjusted for mother’s BMI, the infant’s age in days, the mother’s age, the mother’s energy intake, and secretor status.

To validate the assumptions underlying mixed-effects linear regression models, we examined the residual structure and error distribution using Q-Q plots, scatterplots of errors versus fitted values, and histograms of residuals. Q-Q plots confirmed the normality of residuals with minor deviations, while histograms revealed a symmetrical distribution of residuals, verifying that the normality of errors assumption was met. Scatterplots indicated that the correct form was specified; however, they also revealed a slight qualitative deviation from homoscedasticity. To ensure the robustness of our model, we performed a sensitivity analysis by computing the Z-score of each residual and re-running analysis after removing observations with a residual Z-score greater than 2. After removing these observations, residual analysis plots revealed no evidence of heteroscedasticity. Moreover, the results of the sensitivity analysis were nearly identical to the full results: there were no changes in significance, and the effect sizes of significant terms did not change by more than 10%. Taken in full, this sensitivity analysis indicates that the slight deviation from homoscedasticity is of no concern. Consequently, the results from the full dataset are reported below.

To maintain the readability of our manuscript, we have included an appendix that details the results of all assumption tests (Figure S1). The Supplementary Materials includes diagnostic plots for each test performed, ensuring the full transparency of our analytical procedures. The statistical analysis was performed in R (Version 4.1.3) [28] and Python (Version 3.11.5) [29], and a significance level of 0.05 was utilized to determine the presence of statistically significant associations. We utilized adjusted *p*-values using the Benjamini–Hochberg method for the FDR with a significance level of 0.05.

## 3. Results

### 3.1. Description of Participants

A total of 407 individuals were assessed for eligibility with 214 ultimately enrolled in the study and 210 completing the 1-month visit. Due to participants moving away, declining to participate, or not responding to our contact efforts, we had 131 complete the 6-month visit and 84 complete the 12-month visit. Table 1 shows the characteristics of mother–infant dyads. Mothers were on average 28.9 ± 6.1 years old at baseline with a mean pre-pregnancy BMI of 28.6 kg/m^2^. At baseline, women were breastfeeding on average eight times per day, with 74% of mothers breastfeeding exclusively (exclusive breastfeeding was defined as the infant receiving only breast milk and no other foods or liquids, including water). As expected, mothers were approximately 1 year older at the 12-month visit, and the number of breastfeeding events decreased to five and four events per day at the 6-month and 12-month time points, respectively. Total energy intake, SES index, pre-pregnancy BMI, and breastfeeding exclusivity remained similar at the 6-month and 12-month time points as compared to baseline.

### 3.2. Maternal Dietary Intake at Baseline, 6 and 12 Months

Table 2 presents the observed differences in maternal macronutrient and micronutrient intakes at the baseline, 6-month, and 12-month time points. This analysis is conducted for descriptive purposes, offering a snapshot of intake variations at these intervals. Comparisons between the baseline, 6-month, and 12-month time points unveiled significant differences in some of the maternal macronutrient and micronutrient intakes over time (Table 2). While protein and fat intake did not significantly change over the 12-month study period, the mean maternal carbohydrate intake significantly decreased from 238.8 g (±87.8) at baseline to 219.1 g (±71.8) at 6 months and 210.4 g (±65.9) at 12 months (*p* < 0.04). Additionally, there was a decrease in total sugar intake (*p* < 0.01). The consumption of sugar-sweetened beverages (SSBs) plus juice exhibited significant changes across the three time points (*p* < 0.01). At baseline, participants reported a mean intake of 2.4 servings per day (± 2.7) of SSBs plus juice. However, after 6 months and 12 months, this intake increased from baseline to 5.4 servings per day (± 3.9) and 4.2 servings per day (± 3.5), respectively. There were no significant changes in total fiber, soluble fiber, insoluble fiber, added sugar, free sugar, SSBs excluding juice, or SSBs excluding juice and dairy intakes over time (p_All_ > 0.06).

Significant differences in mean vitamin and mineral intake were observed across the study’s three time points (Table 2). At baseline, the mean daily intakes of vitamins A, C, D, and niacin were 660.5 micrograms (±389.7), 148.8 milligrams (±94.9), 11.2 micrograms (±7.7), and 31.9 milligrams (±13.9), respectively. The maternal intake of vitamins A, C, D, and niacin significantly decreased over the 12-month study period (p_All_ ≤ 0.01). By the 12-month study visit, the mean values for vitamins A, C, D, and niacin decreased to 496.4 micrograms (±232.1), 121.5 milligrams (±178.2), 8.5 micrograms (±12.2), and 26.4 milligrams (±15.6), respectively. Similarly, there were significant decreases in the intakes of calcium, iron, zinc, and potassium (p_All_ < 0.04). At baseline, the mean daily intake of calcium, iron, zinc, and potassium was 1023.9 milligrams (±528.4), 42.3 milligrams (±43.7), 22.3 milligrams (±13.9), and 2403.1 milligrams (±850.8), respectively. After 12 months, calcium, iron, zinc, and potassium intake decreased to 842.3 milligrams (±403.5), 19.5 milligrams (±18.2), 15.9 milligrams (±14.1), 2152 milligrams (±639.9), respectively. There were no other significant changes in assessed vitamins and mineral intake over the 12-month study period (p_All_ > 0.05).

### 3.3. Cross-Sectional Association of Maternal Macronutrient Intake and HMO Profile

**Figure 1:**
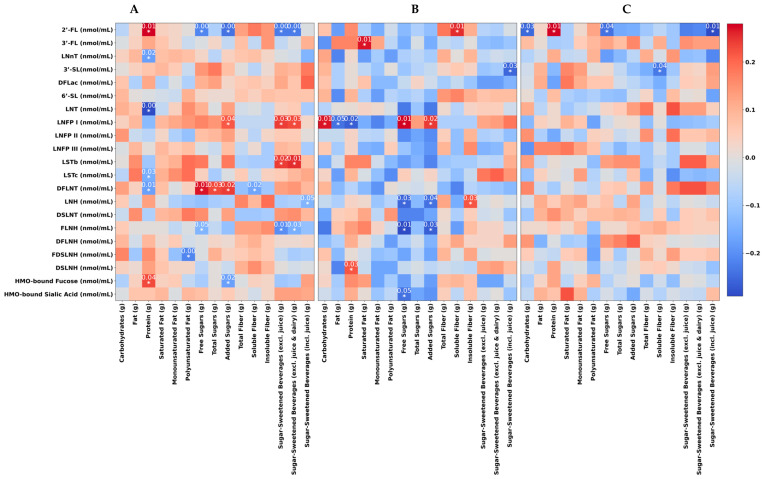
Heatmaps showing the results of the partial correlation analysis between maternal micronutrient intake with HMO composition at infant 1 month (**A**), 6 months (**B**), and 12 months (**C**) of age. Heatmaps display correlation strength (rho values) with blue representing negative correlations and red signifying positive correlations. Asterisks indicate significant correlations with *p*-values from a partial correlation analysis that was adjusted for the mother’s BMI, the infant’s age in days, the mother’s age, the mother’s energy intake, and secretor status. All *p*-values are adjusted for multiple comparisons using the Bonferroni correction.

Partial correlation analysis revealed several associations between maternal macronutrients and HMO composition, with significant outcomes displayed by heatmaps (Figure 1). Our analysis revealed that higher maternal fat intake, particularly total, monounsaturated, and polyunsaturated fats, was significantly associated with lower concentrations of certain HMOs in human milk at 6 and 12 months postpartum. Specifically, a negative correlation between maternal polyunsaturated fat intake and the HMO FDSLNH (rho = −0.20, *p* < 0.01) was observed at 1 month. In addition, higher maternal total fat intake exhibited significant negative correlations with LNFPI (rho = −0.18, *p* = 0.05) at 6 months.

Maternal protein intake was also associated with several HMOs across the study time points. Specifically, at 1 month, maternal protein intake was positively correlated with 2′-FL (rho = 0.19, *p* = 0.01) and HMO-bound fucose (rho = 0.15, *p* = 0.04) and negatively associated with LnNT (rho = −0.17, *p* = 0.02), LNT (rho = −0.26, *p* < 0.01), and DFLNT (rho = −0.17, *p* = 0.01). At 6 months, maternal protein intake was positively associated with DSLNH (rho = 0.19, *p* = 0.03) and negatively associated with LNFP I (rho = −0.20, *p* = 0.02). At 12 months, maternal protein intake was positively correlated with 2′-FL (rho = 0.28, *p* = 0.01).

Maternal dietary sugar intake was correlated with several HMOs. At 1 month, the HMO 2′-FL exhibited a negative correlation with several measures of sugar consumption including free sugar (rho = −0.20, *p* < 0.01), added sugar (rho = −0.22, *p* < 0.01), and SSBs (rho = −0.21, *p* < 0.01), suggesting that higher sugar consumption was associated with reduced levels of 2′-FL in human milk. Consistently, SSB intake was also negatively associated with the concentrations of the HMOs FLNH (rho = −0.19, *p* = 0.01), LNH (rho = −0.14, *p* = 0.04). Conversely, free sugar, added sugar as well as total sugar showed a positive correlation with the HMO DFLNT (rho = 0.18, *p* = 0.01; rho = 0.16, *p* = 0.02 and rho = 0.15, *p* = 0.03, respectively). Furthermore, at 6 months, there was a negative association between the concentration of the HMO LNH and free sugar and added sugar (rho = −0.19, *p* = 0.03 and rho = −0.18, *p* = 0.04, respectively). There was also a positive correlation between the HMO LNFP I and both free sugar and added sugar (rho = 0.24, *p* = 0.01; and rho = 0.21, *p* = 0.02, respectively). Further, SSB intake and 3′-SL were negatively correlated (rho = −0.19, *p* = 0.01) at the 6-month time point. At 12 months, a higher consumption of free sugar and SSBs was negatively correlated with 2′-FL (rho = −0.24, *p* = 0.04 and rho = −0.29, *p* = 0.01, respectively).

Fiber intake was another dietary factor that was correlated with HMO concentrations. Soluble fiber intake was negatively correlated with DFLNT at 1 month (rho = −0.16, *p* = 0.02). Maternal soluble fiber intake displayed a positive correlation with 2′-FL at 6 months (rho = 0.22, *p* < 0.01), indicating a potential link between higher fiber consumption and enhanced levels of 2′-FL within human milk.

### 3.4. Cross-Sectional Association of Maternal Micronutrient Intake and HMO Profile

**Figure 2:**
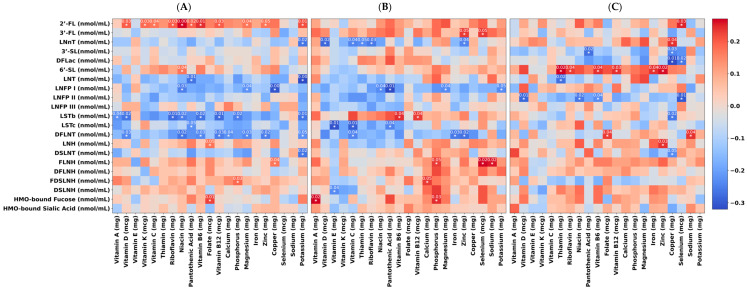
Heatmaps of the results of the partial correlation analysis between maternal micronutrient intake with HMO composition at infant 1 month (**A**), 6 months (**B**), and 12 months (**C**) of age. Heatmaps display correlation strength (rho-values) with blue representing negative correlations and red signifying positive correlations. Asterisks indicate significant correlations with *p*-values from a partial correlation analysis that was adjusted for the mother’s BMI, the infant’s age in days, the mother’s age, the mother’s energy intake, and secretor status. All *p*-values are adjusted for multiple comparisons using the Bonferroni correction.

Maternal micronutrient intake was associated with HMO composition throughout the 12-month study (Figure 2). For example, the intake of B vitamins, except for thiamin and folate, exhibited positive correlations with 2′FL. Additionally, the intake of vitamins D, C, and K showed positive correlations with the HMO 2′-FL, exclusively at the 1-month time point. At 1 month, zinc (rho = 0.14, *p* = 0.04) and potassium (rho = 0.17, *p* = 0.01) showed a positive correlation with 2′-FL. Vitamin D was negatively correlated with LNnT (rho = −0.12, *p* = 0.02) at 6 months. At 12 months, vitamin D (rho = −0.28, *p* = 0.01), niacin (rho = −0.26, *p* = 0.02), vitamin B6 (rho = −0.23, *p* = 0.04), and selenium (rho = −0.30, *p* = 0.01) were all negatively correlated with LNFPII.

### 3.5. Longitudinal Associations of Maternal Diet with the HMOs 2′-FL, 3′-FL, and 3′-SL

Several diet–HMO relationships were identified in a longitudinal analysis of select dietary components and the HMOs of interest (Table 3). The dietary predictor variables were selected based on whether their concentrations significantly changed over time in our study population (Table 2). The HMOs 2′-FL, 3′-FL, and 3′-SL were selected for this analysis based on historical evidence that these are the primary HMOs to shift in concentration over time [18]. A number of nutrients exhibited significant associations with 2′-FL. For example, maternal soluble fiber intake was positively associated with 2′-FL levels. Specifically, for each gram increase in soluble fiber intake, there was a 94.789 nmol/mL increase in 2′-FL concentration in human milk, corresponding to a substantial increase in 2′-FL levels (β = 94.789, 95% CI 25.710, 163.868], *p* < 0.01). Additionally, the coefficient for niacin (mg) is 31.355, which suggests that holding all other factors constant, for every mg increase in niacin intake, there was a 31.355 nmol/mL increase in 2′-FL concentration (β = 31.355, 95% CI [1.672, 61.038], *p* = 0.03). Conversely, zinc intake exhibited a significant negative association with 2′-FL levels. The results showed that for each mg increase in zinc intake, there was a decrease of 40.19 nmol/mL in 2′-FL concentration (β = −40.196, 95% CI [−79.614, −0.778], *p* = 0.04). There were no identified longitudinal associations between the maternal dietary nutrients of interest and the HMOs 2′-FL or 3′-SL over time.

## 4. Discussion

In this paper, we examined associations between maternal dietary intake and HMO composition in human milk obtained from Latino mothers during the first year postpartum. Our results indicate that maternal macronutrient and micronutrient intake are associated with HMO abundances, both cross-sectionally and longitudinally, emphasizing the importance of maternal dietary choices in shaping the changing composition of HMOs.

Our results revealed that maternal carbohydrate intake during lactation is associated with HMO levels [30]. Specifically, we found that the intake of sugar, including free sugar, and added sugar were negatively correlated with 2′-FL and positively correlated with DFLNT at the 1-month time point. Additionally, sugar intake was negatively correlated with LNH and positively correlated with LNFP I at the 6-month time point. Further, our results indicate that sugar intake in the form of SSBs may be especially influential as our analysis reveals that SSB consumption negatively influenced the concentrations of fucosylated HMOs, specifically, 2′-FL, LNH, and FLNH at the 1-month time point. These findings support two previous single-blinded cross-over dietary intervention studies conducted by Seferovic et al. [20] which examined how maternal diet affects human milk composition. In the first study, lactating women consumed liquid drinks containing either glucose or galactose as the exclusive carbohydrate source. They observed that the total HMO-bound fucose concentration decreased with glucose compared to galactose, although individual HMOs exhibited varying responses [20].

Furthermore, our investigation builds on the second study by Seferovic et al., which assigned lactating women to either high-fat or high-carbohydrate diets for eight days. They found that the total HMO-bound sialic acid concentration decreased with the high-fat diet, although individual sialylated HMOs did not show significant differences between the diets. These results may be attributed to the study being underpowered to detect differences in individual HMOs [20]. Our findings align with the broader observation that maternal dietary fat intake influences the HMO profile. Specifically, we found a correlation between an increased maternal intake of polyunsaturated fats and decreased levels of certain HMOs, such as FDSLNH at 1 month, and a negative correlation between total fat consumption and LNFP I at 6 months. These observations imply that a higher fat diet may influence lower HMO concentrations.

Our study also explored the associations of the maternal intake of fiber on HMO abundances. We found that the maternal intake of soluble fiber exhibited positive correlations with specific HMOs, particularly 2′-FL at the 6-month time point. In contrast, our study showed a negative correlation between soluble fiber and DFLNT. These findings build upon earlier research, which found that specific HMOs in women’s milk were linked to distinct nutrient patterns, including insoluble and soluble fiber intake. For example, Selma-Royo et al. found that FLNH and FDSLNH were positively associated with maternal intakes of insoluble fiber, cellulose, hemicellulose, and polyphenols [31]. Overall, these results indicate that dietary fibers differentially influence the HMO profile. Our study suggests both positive and negative associations between soluble fiber intake and specific HMOs.

In our analysis, we identified several associations between individual vitamins and HMO composition. Specifically, we discovered positive associations between vitamin intake and fucosylated HMOs. For instance, 2′-FL was positively correlated with vitamins D, K, C, and various B vitamins. Further, our longitudinal analysis revealed a significant association between niacin intake and 2′-FL. For every unit increase in niacin intake, there was a 31.35 unit increase in 2′-FL concentration. These findings align with a similar observational study conducted in China, which utilized repeated Food Frequency Questionnaires (FFQs) over the lactation periods (0–400 days). In that study, significant positive correlations were observed between certain micronutrients and specific HMOs. Specifically, vitamins A, C, B1, and B2 were found to be positively associated with fucosylated HMOs. Our results paired with the results by Li et al. indicate that vitamin supplements could potentially be a favorable nutritional approach to enhance HMO levels in the milk of lactating mothers [32,33].

Our study revealed intriguing associations between metals and individual HMOs across various time points. Initially, at 1 month, we observed a positive correlation between zinc and 2′-FL, indicating a potential link between maternal zinc intake and the presence of 2′-FL during early lactation. This observation is consistent with the previous literature emphasizing the role of metals in HMO biosynthesis [33,34]. However, upon considering the longitudinal trajectory of zinc intake, which significantly decreased from 1 month to 12 months postpartum, a different trend emerged. Contrary to our initial cross-sectional observation, the longitudinal analysis revealed a negative correlation between zinc and 2′-FL over time. This underscores the importance of considering longitudinal changes in dietary intake when assessing associations with HMO concentrations. It also raises intriguing questions about the dynamic interplay between maternal diet, HMO production, and lactation duration. Furthermore, at 1 month, copper was positively correlated with FLNH. By 6 months, zinc showed a positive correlation with 3′-FL, while selenium was positively associated with both 3′-FL and FLNH. At 12 months, selenium exhibited a positive correlation with 2′-FL, and LNnt was positively correlated with copper. Additionally, at 6 months, positive correlations were found for both 6′-SL and iron, as well as for LNH and zinc.

In a study conducted in China, researchers investigated the correlation between maternal dietary intake and breastmilk human milk oligosaccharide (HMO) concentrations across all lactation periods (0–400 days) among lactating women [33]. They found that the dietary intake of metal elements was significantly correlated with breastmilk HMO concentrations. Notably, they observed that metals emerged as the most influential predictive factor for the cumulative concentration of all HMOs in the mixed-effects model [33]. However, in contrast to our research, their findings indicated that metals did not significantly predict individual HMOs, implying that metals may potentially stimulate a broad spectrum of HMO concentrations, possibly operating via a distinct mechanism compared to other nutrients that exhibit associations with specific individual HMOs [33]. These results align with our own findings, further emphasizing the importance of maternal dietary factors in influencing HMO production.

While our results add evidence of the role of maternal nutrition in shaping the HMO profile, the mechanisms for this relationship remain unclear. This is partially because HMOs are unique compounds present only in milk, and the mechanisms underlying the production of HMOs within the body remain largely unknown. It is hypothesized that HMOs are formed by the elongation of lactose with other monosaccharides, a process catalyzed by glycosyltransferases in the mammary gland [19,35]. Thus, we postulate that in conditions of adequate nutrition, glycosyltransferase activity may significantly contribute to elevated HMO levels. Several vitamins, including vitamin B1 and vitamin B2, act as coenzymes, while minerals are crucial for enzymatic function, either incorporating them into the active site or relying on them for enzyme activation [34]. For instance, the impact of vitamins D, C, K, and minerals such as zinc and potassium on 2′-FL concentration involves distinct mechanisms: vitamin D is associated with the modulation of gene expression for glycosyltransferases; vitamin C enhances enzyme activity by acting as a cofactor; vitamin K is involved in the carboxylation of proteins related to enzymatic processes; zinc serves as a direct cofactor for glycosyltransferases, facilitating specific reactions for 2′-FL production; and potassium contributes to creating favorable intracellular conditions for HMO synthesis. Exploring how these nutrients enhance glycosyltransferase activity and uncovering their mechanisms of action are potential areas for future research.

This study is the first to comprehensively investigate associations between maternal dietary intake and the composition of quantitated HMOs in mature human milk samples in a Latina population. While we investigate specific previous studies and their findings in the Section 4, it is important to note here that our research builds upon and expands the existing body of knowledge by focusing on dietary impacts on HMO profiles in this particular demographic group. A distinctive strength of our study is the utilization of multiple time points with three-day dietary recalls, providing a unique perspective on the changing HMO profiles throughout lactation. This approach sets us apart from previous studies, which primarily relied on single dietary recalls in smaller sample sizes [36]. Additionally, our study reports on both macronutrient and micronutrient intake, while much of the previous work focused solely on macronutrient intake. We are also one of the first studies to report associations between maternal SSB intake with the HMO profile.

In our analysis, certain variables showed minor deviations from the ideal of normality. However, these deviations were within acceptable limits and did not compromise the integrity of our regression models [37,38]. Our supplementary use of the Spearman correlation addresses the robustness of our findings, mitigating concerns regarding non-normal distributions. Further discussing our limitations, while our study’s focus on an ethnically homogenous population may limit the generalizability of our findings, it also serves as a strength allowing for the investigation of a traditionally under-represented population and providing valuable insights into the health outcomes of this specific demographic. Additionally, since this study was observational, we cannot infer whether the associations are indicative of a causal relationship. It is essential to acknowledge the potential presence of unmeasured or unaccounted-for confounders, which can introduce bias into our findings. Another limitation of our study is the reliance on self-reported dietary recalls which can be subject to recall bias and may not reflect the precise details of participants’ dietary intake. Despite this limitation, self-reported recalls remain a common method, and we took steps to minimize potential biases.

Overall, our results support the role of maternal macronutrient and micronutrient intake in shaping the HMO profile. This is important as growing evidence supports the role of HMO intake during this critical window of infant growth and development [36]. For example, our study shows that reducing sugar intake and increasing soluble fiber may increase 2′-FL levels, which may potentially impact brain development and improve infant cognitive development [10]. In addition, 2′-FL exerts influence on host epithelial cell immune responses. It possesses the ability to selectively block the attachment of pathogenic bacteria and viruses to the gut epithelium, thereby acting as a protective barrier against the development of diseases [39]. Furthermore, 2′-FL may enhance the integrity of the gastrointestinal barrier and fosters a gut microbiome enriched with bifidobacteria, which may not only guard against infections but also fortify the epithelial barrier while generating immunomodulatory metabolites [13]. These findings emphasize the role of diet and breastfeeding in early cognitive growth and immune system maturation through the effects of HMOs on infant gut microbiome composition. Therefore, altering HMO composition through maternal dietary intake may benefit infant outcomes with beneficial effects extending well beyond those early years. Future investigations should expand on our results and the results of others to explore causal relationships between identified nutritional factors and HMOs of interest.

## 5. Conclusions

This study provides specific evidence that maternal macro- and micronutrient intake is associated with variations in the HMO profile in a cohort of Latino mothers from Southern California over a 12-month postpartum period. Notably, our findings demonstrate a negative correlation between the maternal intake of free sugar, added sugar, and sugar-sweetened beverages and the level of the most abundant HMO, 2′-FL, particularly at 1 month postpartum. This suggests that higher sugar consumption could be linked to lower 2′-FL levels which could be detrimental for infant development. A positive correlation emerged between the intake of specific vitamins and fucosylated HMOs. Notably, vitamins D, K, C, and B showed a significant association with increased levels of 2′-FL. The longitudinal analysis indicated that increased maternal soluble fiber intake was linked to higher 2′-FL levels in human milk, whereas higher zinc intake was associated with a reduction in 2′-FL concentration. Additionally, a positive correlation was observed between niacin intake and 2′-FL levels. Therefore, the results suggests that the HMO profile may be modulated through maternal dietary interventions, which could have lasting impacts on infant health and development, especially as it relates to infant microbiota development, immune system maturation, and cognitive development. However, further studies including maternal dietary intervention during lactation are needed to establish whether there is a causal relationship between maternal diet and the HMO profile. Additionally, research is needed to determine whether similar diet–HMO relationships exist among a diverse range of populations that vary by ethnic background and diet quality.

## Figures and Tables

**Table 1:** Demographic characteristics, anthropometric measurements, and HMO distribution in Latino mothers.

	1 Month n = 210	6 Months n = 131	12 Months n = 84	*p*-Value
**Mothers**				
Maternal Age (years)	28.9 ± 6.1	29.5 ± 6.2	29.9 ± 6.3	0.39
Pre-pregnancy maternal BMI (kg/m^2^)	28.6 ± 5.9	28.5 ± 5.8	27.7 ± 5.2	0.46
Maternal BMI (kg/m^2^)	30.3 ± 5.2	30.4 ± 5.5	29.2 ± 5.4	0.19
Breastfeeding/day	8.2 ± 2.7	5.4 ± 3.9	4.2 ± 3.5	<0.00 *
Secretors, n (%)	183 (87.56)	114 (87.02)	76 (89.41)	<0.00 *
**Infants**				
Age (days)	32.6 ± 4.6	184.9 ± 9.2	367.3.3 ± 8.3	<0.00 *
**HMO distribution (nmol/mL)**				
2′-Fucosyllactose (2′-FL)	6262 ± 3634.4	6515.5 ± 3875.1	6322.7 ± 3209.5	0.819
3′-Fucosyllactose (3′-FL)	941.8 ± 1375.1	2281.2 ± 2299.2	3623.2 ± 2569.9	<0.01 *
3′-Sialyllactose (3′-SL)	547.5 ± 507.9	739.6 ± 414.4	905 ± 654.7	<0.01 *
6′-Sialyllactose (6′-SL)	1015.9 ± 338.6	280.2 ± 191.5	147.9 ± 126.9	<0.01 *
Difucosyllactose (DFLac)	423 ± 318.2	606.3 ± 424.8	662.9 ± 408.8	<0.01 *
Difucosyllacto-N-hexaose (DFLNH)	148.7 ± 130.6	64.3 ± 57	62.6 ± 53.2	<0.01 *
Difucosyllacto-N-tetrose (DFLNT)	1382.7 ± 753.3	1155.4 ± 696.1	1065.2 ± 586.3	<0.01 *
Disialyllacto-N-hexaose (DSLNH)	251.4 ± 108.9	72.8 ± 62.3	36.1 ± 28.4	<0.01 *
Disialyllacto-N-tetraose (DSLNT)	398.4 ± 191.6	292 ± 177	257.8 ± 140.7	<0.01 *
Fucodisialyllacto-N-hexaose (FDSLNH)	171.7 ± 190.6	217.9 ± 149.1	153.4 ± 89.1	<0.01 *
Fucosyllacto-N-hexaose (FLNH)	158.7 ± 112.1	75.6 ± 73.9	68.3 ± 47.4	<0.01 *
Lacto-N-fucopentaose I (LNFP-I)	1359.6 ± 1189.8	500.8 ± 539	476.6 ± 569.1	<0.01 *
Lacto-N-fucopentaose II (LNFP-II)	970.1 ± 608.6	1325 ± 683	1208.6 ± 581.5	<0.01 *
Lacto-N-fucopentaose III (LNFP-III)	64.9 ± 39.4	69.2 ± 59.4	54.4 ± 28.6	0.12
Lacto-N-hexaose (LNH)	102 ± 63.7	74.9 ± 55.6	71.6 ± 58.7	<0.01 *
Lacto-N-neotetraose (LNnT)	565.3 ± 337.4	339.4 ± 229.1	251.4 ± 125.1	<0.01 *
Lacto-N-tetraose (LNT)	1132.5 ± 692.2	734.7 ± 532.9	746.3 ± 407.4	<0.01 *
Sialyllacto-N-tetraose b (LSTb)	91.4 ± 65.5	80.7 ± 58.6	80.6 ± 63.7	0.218
Sialyllacto-N-tetraose c (LSTc)	312.4 ± 138.1	84.9 ± 88.1	62 ± 80.5	<0.01 *
HMO-bound Fucose	13,837.6 ± 3328.7	14,637.2 ± 4181.5	15,488.5 ±3205	<0.01 *
HMO-bound Sialic Acid	3610.2 ± 840	2350.7 ± 804.3	2090.2 ± 721.9	<0.01 *

Values are mean ± standard deviation (SD) or frequency (percentage) for categorical variables. Normality and homoscedasticity tests were run for all variables. ANOVA was utilized for normally distributed and homogeneous variables, while Kruskal–Wallis test was employed for non-normally distributed and heterogeneous variables. Statistically significant differences between time points are indicated by asterisks (*). Abbreviations: BMI: body mass index.

**Table 2:** Maternal nutrient intake at infant 1, 6, and 12 months of age.

Dietary Variables	1 Month n = 210	6 Months n = 131	12 Months n = 84	*p*-Value
Energy	1800 ± 590	1770 ± 510	1701 ± 460	0.39
**Macronutrients**				
Protein (g)	78.7 ± 25.2	79.9 ± 24.2	76.4 ± 21.2	0.58
Fat (g)	62 ± 26.5	66.3 ± 24.3	64 ± 23.6	0.30
Saturated fat (g)	20.5 ± 10.2	20.9 ± 8.5	20.3 ± 7.9	0.90
Monounsaturated fat (g)	21.1 ± 9.6	23.3 ± 9	22.6 ± 9.1	0.09
Polyunsaturated fat (g)	14.2 ± 7.1	15.7 ± 8	14.8 ± 6.9	0.21
Carbohydrate (g)	238.8 ± 87.8	219.1 ± 71.8	210.4 ± 65.9	0.04
Total fiber (g)	18.6 ± 7.2	17.9 ± 7	18.2 ± 7.6	0.68
Insoluble fiber (g)	12.6 ± 5.1	12.4 ± 5.8	12.8 ± 6.1	0.86
Soluble fiber (g)	5.9 ± 3.1	5.3 ± 2	5.2 ± 2.1	0.18
Total Sugar (g)	103.2 ± 51.7	88.9 ± 39.8	80.6 ± 38.1	<0.01 *
Added sugar (g)	59.2 ± 40.8	54.7 ± 33.7	51.4 ± 34	0.23
Free sugar (g)	63.4 ± 41.2	57 ± 33.6	52.7 ± 33.9	0.06
**Sugary Sweetened Beverage (SSB)**				
SSB excluding juice (servings/day)	0.9 ± 1	0.9 ± 9.9	1 ± 1.3	0.76
SSB excluding juice and dairy (servings/day)	0.8 ± 1	0.9 ± 0.9	1 ± 1.3	0.60
SSB plus juice (servings/day)	2.4 ± 2.7	5.4 ± 3.9	4.2 ± 3.5	<0.01 *
**Micronutrients**				
Vitamin A (mcg)	660.5 ± 389.7	533.5 ± 268.7	496.4 ± 232.1	<0.01 *
Vitamin D (mcg)	11.2 ± 7.7	8.1 ± 8.5	8.5 ± 12.2	<0.01 *
Vitamin E (mg)	6.6 ± 3.3	7.3 ± 4.1	7 ± 3.9	0.21
Vitamin K (mcg)	76.7 ± 66.1	85.6 ± 65.5	96.1 ± 120.9	0.15
Vitamin C (mg)	148.8 ± 94.9	116.1 ± 78.8	121.5 ± 178.2	0.01 *
Thiamin (mg)	2.5 ± 1.6	2.3 ± 4.5	2.4 ± 3.2	0.88
Riboflavin (mg)	2.8 ± 1.8	2.6 ± 4.5	2.6 ± 3.3	0.74
Niacin (mg)	31.9 ± 13.9	27.8 ± 13.3	26.4 ± 15.6	<0.01 *
Pantothenic acid (mg)	5.2 ± 2.5	5.9 ± 5.7	6.3 ± 6.5	0.13
Vitamin B6 (mg)	3.4 ± 2.4	3.1 ± 4.9	3 ± 3.7	0.55
Folate (mcg)	368.4 ± 185.4	335.4 ± 142.9	323.3 ± 138.7	0.05
Vitamin B12 (mcg)	8.7 ± 7.1	14.2 ± 87.4	6.8 ± 7.7	0.48
Calcium (mg)	1023.9 ± 528.4	834.1 ± 430.2	842.3 ± 403.5	<0.01 *
Phosphorus (mg)	1307.7 ± 462.8	1229.4 ± 395.7	1187.7 ± 308.2	0.18
Magnesium (mg)	291.1 ± 96.5	277.9 ± 85.9	272.7 ± 82.9	0.23
Iron (mg)	42.3 ± 43.7	21.7 ± 18.9	19.5 ± 18.2	<0.01 *
Zinc (mg)	22.3 ± 13.9	16.1 ± 11.4	15.9 ± 14.1	<0.01 *
Copper (mg)	1.1 ± 0.4	1.2 ± 0.6	1.2 ± 1	0.47
Selenium (mcg)	107.6 ± 37.4	112 ± 39.6	108.3 ± 40.8	0.58
Sodium (mg)	2562.9 ± 930.6	2689.5 ± 1031.5	2545.4 ± 844.3	0.41
Potassium (mg)	2403.1 ± 850.8	2296 ± 734.8	2152 ± 639.9	0.08 *
Manganese (mg)	2.9 ± 1.3	2.7 ± 1.2	2.6 ± 1.4	0.22

Values are mean ± standard deviation (SD). Normality and homoscedasticity tests were run for all variables. ANOVA was utilized for normally distributed and homogeneous variables, while Kruskal–Wallis test was employed for non-normally distributed and heterogeneous variables. Statistically significant differences between time points are indicated by asterisks (*). Abbreviations: SSB: sugar-sweetened beverage.

**Table 3:** Mixed linear regression results for dietary components and HMO composition (n = 210).

	Beta-Coefficient	95% CI	*p*-Value
**2′-FL**			
Carbohydrate (g)	0.743	[−9.635, 11.121]	0.88
Total Sugar (g)	−6.597	[−18.626, 5.432]	0.28
Soluble fiber (g)	94.789	[25.710, 163.868]	<0.01 *
SSB plus juice (servings/day)	1.400	[−118.417, 121.217]	0.98
Vitamin A (mcg)	−0.068	[−1.049, 0.913]	0.89
Vitamin C (mg)	1.586	[−2.249, 5.421]	0.41
Niacin (mg)	31.355	[1.672, 61.038]	0.03 *
Folate (mcg)	0.228	[−1.766, 2.223]	0.82
Calcium (mg)	0.720	[−0.155, 1.595]	0.10
Phosphorus (mg)	0.387	[−0.897, 1.672]	0.55
Iron (mg)	2.449	[−5.797, 10.696]	0.56
Zinc (mg)	−40.196	[−79.614, −0.778]	0.04 *
Potassium (mg)	−0.070	[−0.696, 0.555]	0.82
**3′-FL**			
Carbohydrate (g)	−2.107	[−9.249, 5.036]	0.56
Total Sugar (g)	−5.501	[−13.205, 2.203]	0.21
Soluble fiber (g)	16.031	[−9.126, 41.188]	0.68
SSB plus juice (servings/day)	29.473	[−53.852, 112.799]	0.48
Vitamin A (mcg)	0.487	[−0.219, 1.193]	0.17
Vitamin C (mg)	2.885	[−0.585, 6.356]	0.10
Niacin (mg)	−10.136	[−32.644, 12.373]	0.37
Folate (mcg)	−0.036	[−1.449, 1.375]	0.95
Calcium (mg)	−0.097	[−0.997, 0.803]	0.91
Phosphorus (mg)	0.751	[−0.169, 1.671]	0.10
Iron (mg)	−0.879	[−7.462, 5.704]	0.78
Zinc (mg)	1.818	[−27.255, 30.891]	0.90
Potassium (mg)	−0.258	[−0.695, 0.179]	0.24
**3′-SL**			
Carbohydrate (g)	0.220	[−1.919, 2.358]	0.84
Total Sugar (g)	−2.039	[−4.769, 0.690]	0.14
Soluble fiber (g)	2.300	[−21.747, 26.347]	0.85
SSB plus juice (servings/day)	17.281	[−9.197, 43.758]	0.20
Vitamin A (mcg)	−0.005	[−0.220, 0.209]	0.96
Vitamin C (mg)	−0.399	[−1.437, 0.639]	0.45
Niacin (mg)	−2.520	[−9.794, 4.755]	0.49
Folate (mcg)	−0.326	[−0.774, 0.122]	0.15
Calcium (mg)	0.022	[−0.169, 0.213]	0.82
Phosphorus (mg)	−0.133	[−0.430, 0.163]	0.37
Iron (mg)	−0.327	[−2.272, 1.618]	0.74
Zinc (mg)	2.010	[−6.894, 10.914]	0.65
Potassium (mg)	0.101	[−0.039, 0.241]	0.15

The table presents the results of mixed linear regression analyses for the relationship between dietary components and HMO composition, based on a dataset of 80 participants with complete diet data at all time points. In the table, beta coefficients, confidence intervals, and *p*-values are reported. A *p*-value less than 0.05 is considered statistically significant and indicated by asterisks (*).

## Data Availability

An anonymized dataset including all data described in the manuscript, code book, and analytic code will be made available upon request to the principal investigator M.I.G.

## Supplementary Materials

The following supporting information can be downloaded at: https://www.mdpi.com/article/10.3390/nu16121795/s1, Figure S1. Diagnostic plots for assessing model fit and residuals. (A) Q-Q plot, scatter plot of residuals vs. fitted values, and histogram of residuals for Model 1. (B) Corresponding plots for Model 2. (C) Similar set of plots for Model 3. Each row of plots pertains to a distinct model, showcasing the respective checks for normality, spread of residuals, and distributional characteristics.
