# Supplementary material for: Maternal Diet Associated with Oligosaccharide Abundances in Human Milk from Latina Mothers
Source: Nutrients. 2024 Jun 7;16(12):1795. doi: 10.3390/nu16121795 (PMC11206877; doi:10.3390/nu16121795)
Supplement: Supplementary file 1 [file nutrients-16-01795-s001.zip › nutrients-3037087-supplementary.pdf]

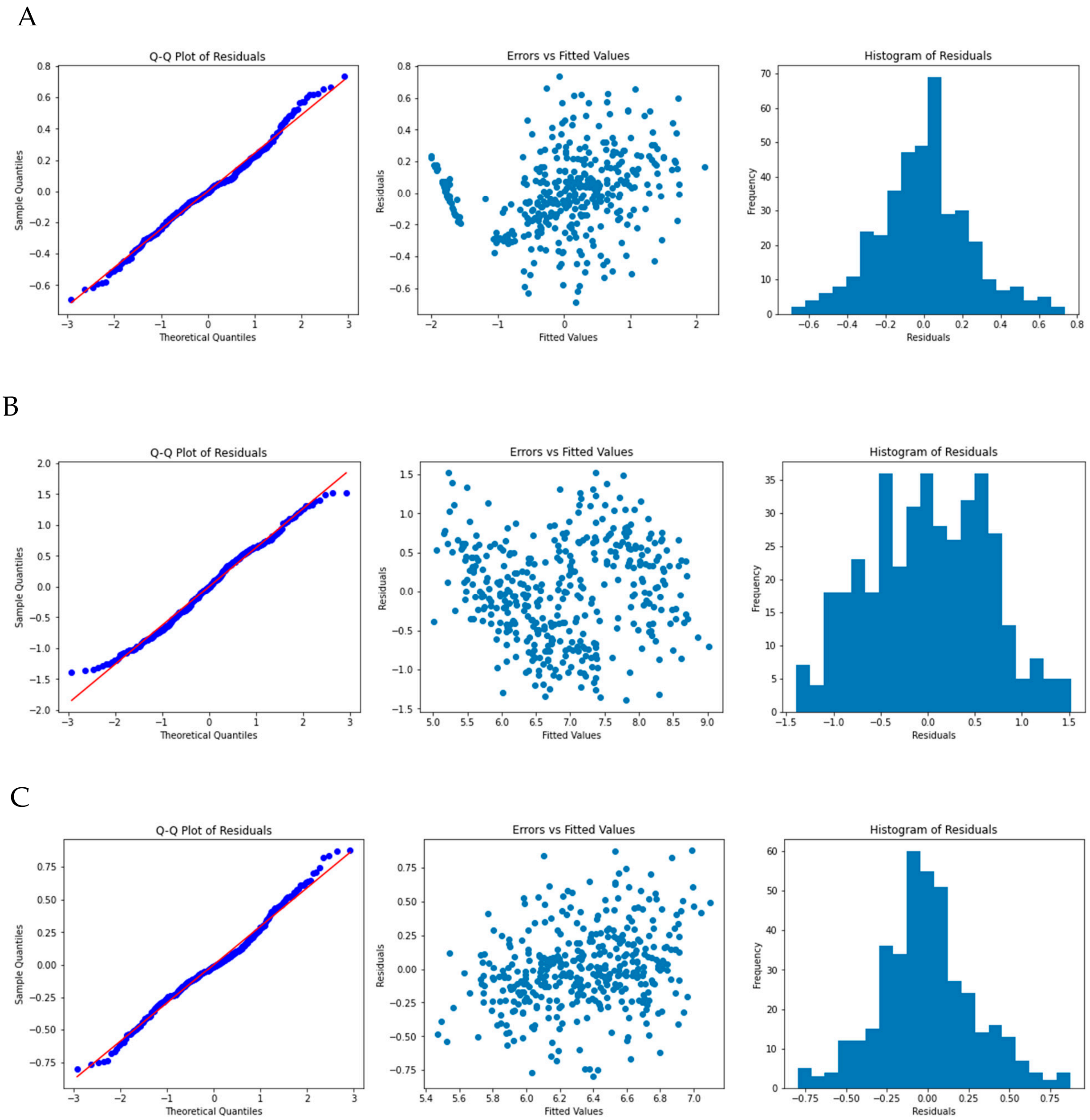

Figure S1. Diagnostic plots for assessing model fit and residuals. (**A**) Q-Q plot, scatter plot of residuals vs. fitted values, and histogram of residuals for Model 1. (**B**) Corresponding plots for Model 2. (**C**) Similar set of plots for Model 3. Each row of plots pertains to a distinct model, showcasing the respective checks for normality, spread of residuals, and distributional characteristics.
